# A New Algorithm for Identifying Genome Rearrangements in the Mammalian Evolution

**DOI:** 10.3389/fgene.2019.01020

**Published:** 2019-10-29

**Authors:** Juan Wang, Bo Cui, Yulan Zhao, Maozu Guo

**Affiliations:** ^1^School of Computer Science, Inner Mongolia University, Hohhot, China; ^2^School of Electrical and Information Engineering, Beijing University of Civil Engineering and Architecture, Beijing, China; ^3^Beijing University of Civil Engineering and Architecture, Beijing Key Laboratory of Intelligent Processing for Building Big Data, Beijing, China

**Keywords:** genome rearrangements, mammal, phylogenetic tree, evolution, algorithm

## Abstract

Genome rearrangements are the evolutionary events on level of genomes. It is a global view on evolution research of species to analyze the genome rearrangements. We introduce a new method called RGRPT (recovering the genome rearrangements based on phylogenetic tree) used to identify the genome rearrangements. We test the RGRPT using simulated data. The results of experiments show that RGRPT have high sensitivity and specificity compared with other tools when to predict rearrangement events. We use RGRPT to predict the rearrangement events of six mammalian genomes (human, chimpanzee, rhesus macaque, mouse, rat, and dog). RGRPT has recognized a total of 1,157 rearrangement events for them at 10 kb resolution, including 858 reversals, 16 translocations, 249 transpositions, and 34 fusions/fissions. And RGRPT has recognized 475 rearrangement events for them at 50 kb resolution, including 332 reversals, 13 translocations, 94 transpositions, and 36 fusions/fissions. The code source of RGRPT is available from https://github.com/wangjuanimu/data-of-genome-rearrangement.

## Introduction

The rapid development of sequencing technologies makes the phylogenetic analysis from the level of whole genome possible. A studied genome is represented as a line of conserved segments (called syntenic blocks). The genome rearrangements of species are changes of syntenic block orderings and losing of sequence blocks. These events include reversal, translocation, transposition, fusion, fission, and so on (Xu et al. 2017; Cheng et al. 2019; Dong et al., 2018). The research on genome rearrangements is mainly three aspects.

One is the computation of evolutionary distance between two species by considering genome rearrangements. Researchers have proposed a lot of metric for measuring the dissimilarity of evolution between species and a large amount of algorithms for computing the metrics. The breakpoint distance is the minimum rearrangement operations transforming one genome to the other genome, which is computed by means of breakpoint graph (Blanchette et al., 1997; Sankoff and Blanchette, 1998). There are lots of algorithms for computing breakpoint distance. In 1995, Hannenhalli and Pevzner put forward an algorithm with O(*n^5^*) time complexity to compute the breakpoint distance just considering reversal events (Hannenhalli and Pevzner, 1999). Later, Kaplan improved the algorithm to time complexity O(*n^5^*) (Kaplan et al., 2000). In 1996, Hannenhalli designed an algorithm with O(*n^3^*) time complexity to compute it by considering translocation events (Hannenhalli, 1995). In 2001, Zhu et al. improved the algorithm to time complexity O(*n^2^logn*) (Zhu and Ma, 2002). And then Zhu et al. devised an algorithm with O(*n^2^*) time complexity (Liu et al., 2004). The DCJ distance is introduced by Yancopoulos et al. (Sophia et al., 2005), which uses the double cut and join (DCJ for short) operation to model rearrangement events, such as reversal, translocation, transposition, fusion, and fission in an unified way. Yancopoulos et al. first propose a method to compute the DCJ distance by considering only translocations and reversals on linear chromosomes (Sophia et al., 2005). Paper (Lu et al., 2006) has proposed an *O(n^2^)* time algorithm to compute the distance by considering the fusions and fissions between circular unsigned chromosomes. Unimog (Hilker et al., 2012) is software for computing DCJ distance which implements lots of algorithms (Erdös et al., 2011; Jakub et al., 2011). SoRT is a tool to compute breakpoint distance and the DCJ distance for linear/circular multi-chromosomal gene orders (Yen-Lin et al., 2010). SCJ distance (Feijão and Meidanis, 2011) is defined using the single cut and join (SCJ for short) operations, which is in analogy to DCJ measure. The distance can be computed by a speedily computable.

Two is the reconstruction of the ancestral gene orders by using the genomes of extant species. Ma et al. (Ma et al., 2006) use maximum parsimony principle to recover reliably ancestral genomes starting from phylogenetic tree and adjacent genes in genome and make the probabilistic reconstruction accuracy analysis for the six mammalian genome (human, mouse, rat, dog, opossum, and chicken) based on the improved Jukes–Cantor model. PMAG utilized the Bayesian theorem in the probabilistic framework to infer ancestral genomes (Yang et al., 2014). Multiple Genome Rearrangements (MGR) recovers the ancestral genome by minimizing the rearrangement distance (Bourque and Pevzner, 2002). Multiple Genome Rearrangements and Ancestors (MGRA) is developed to reconstruct ancestral genomes based on multiple breakpoint graphs and is used to analyze rearrangement evolutionary events of seven mammalian genomes (human, chimpanzee, macaque, mouse, rat, dog, and opossum) (Alekseyev and Pevzner, 2009). Decostar (Duchemin et al., 2017) is a software which reconstructs neighborhood relations of ancestral genes aiming at reconstructing the organization of ancestral genomes.

Three is the recognition of the rearrangement events of existing species. Efficient Method to Recover Ancestral Events (EMRAE) is an algorithm which can recognize rearrangement events in evolution described by phylogenetic tree by means of adjacent genes in genomes (Zhao and Bourque, 2009).

## Materials and Methods

### Preliminaries

A genome is composed of several chromosomes, and each chromosome is an ordering of syntenic blocks. For convenience, each syntenic block is recorded by an integer, so a chromosome is represented by a signed permutation *X*=*c*_1_ *c*_2_⋯*g_n_*, where *c_i_*(1≤*i*≤*n*) is an integer representing a syntenic block, its sign is assigned with the orientation that is either positive (recorded by *c_i_*) or negative (recorded by –*c_i_*). The chromosome *X*=*c*_1_*c*_2_⋯*c_n_* is the same as –*X* = – *c_n_* – *c_n_*_– 1_… – *c*_1_.

A reversal *r* (*i*, *j*) (*i* ≤ *j*) converts chromosome *X*=*c*_1_*c*_2_⋯*c_n_* into a new chromosome *X*'=*c*_1_ *c*_2_⋯−*c_j_*−*c_j_*_–1_⋯−*c_i_*_+1_−*c_i_c _j_*_+1_⋯*c_n_*, where the reversal is from *c_i_* to *c_j_*.

A translocation event breaks two chromosomes into four segments and then reconnects them into two new chromosomes. Given two chromosomes *X* = *X*_1_ *X*_2_ and *Y* = *Y*_1_ *Y*_2_, where *X*_1_=*x*_1_ *x*_2_⋯*x _i_*_–1_,*X*_2_=*x_i_x_i_*_+1_⋯*x _m_*,*Y*_1_=*y*_1_ *y*_2_⋯*y_j_*_–1_, and *Y_2_*=*y _j_y_j_*_+1_⋯*y _n_*, a translocation is represented by *tl*(*i*,*j*). *X*_1_ and *Y*_1_ are exchanged to form two new chromosomes *X*'=*Y*_1_ *X*_2_ and *Y*'=*X*_1_ *Y*_2_, or *X*_1_ and *Y*_2_ are exchanged to form two new chromosomes *X*” = – *Y*_2_ *X*_2_ and *Y*” = *X*_1_ – *Y*_1_. 

A transposition event is to exchange two adjacent fragments on one chromosome into a new chromosome. A transposition is represented by *tp*(*i*, *j*, *k*), i.e., the fragment *c_i_*⋯*c_j_* of one chromosome inserted into after *c_k_*. If *c_k_* is on the same chromosome (*k* > *j* or *k* < *i*), then the transposition *tp*(*i*, *j*, *k*) is called intra-chromosomal; otherwise, it is inter-chromosomal. Given a chromosome *X*=*c*_1_ *c*_2_⋯*c _i_c_i_*_+1_⋯*c _j_*_–1_ *c _j_*⋯*c _k_*⋯*c _n_* and an intra-chromosomal transposition, *X* is converted into *X*'=*c*_1_ *c*_2_⋯*c_k_c_i_c_i_*_+1_⋯*c _j_c_k_*_+1_⋯*c _n_*.

A fusion event is to connect two chromosomes into a new chromosome. The fusion acting on chromosomes *X*_1_ and *X*_2_ is represented by *f u*(*X*_1_, *X*_2_) and forming a new chromosome *X*_1_ *X*_2_ or *X*_1_−*X*_2_. A fission is to split a chromosome into two new chromosomes. A fission acting on the chromosome *X* = *X*_1_ *X*_2_ is represented by *f i*(*X*) and forming two new chromosomes *X*_1_ and *X*_2_ (where *X*_1_ and *X*_2_ are non-empty segments).

An adjacency *a*(*c _i_*,*c _i_*_+1_) of genome *X* is two adjacent integers in one chromosome of *X*. *a*(*c _i_*,*c _i_*_+1_) is the same as *a*(−*c_i_*_+1_,−*c _i_*). For example, all adjacencies on chromosome *X=* 1,234 are *a*(1, 2), *a*(2, 3), and *a*(3, 4). For a set of genomes *S*, an adjacency *a* is effective w.r.t. *S* if it belongs to at least one genome and not all genomes. For example, two uni-chromosomal genomes *G*_1_ and *G*_2_, the chromosome *X=* 1,234 of *G*_1_ and the chromosome *Y=* 1 – 3 − 24 of *G*_2_, then all effective adjacencies w.r.t. *G*_1_ and *G*_2_ are *a*(1, 2), *a*(2, 3), *a*(3, 4), *a*(1, −3), and *a*(−2, 4).

### EMRAE

Given a phylogenetic tree *T* describing the evolution of the genomes *G*, EMRAE first computes all effective adjacencies w.r.t. *G*. Then, it predicts the rearrangement events for each edge of *T* by means of inference rules (will be introduced in the following).

**Figure 1:**
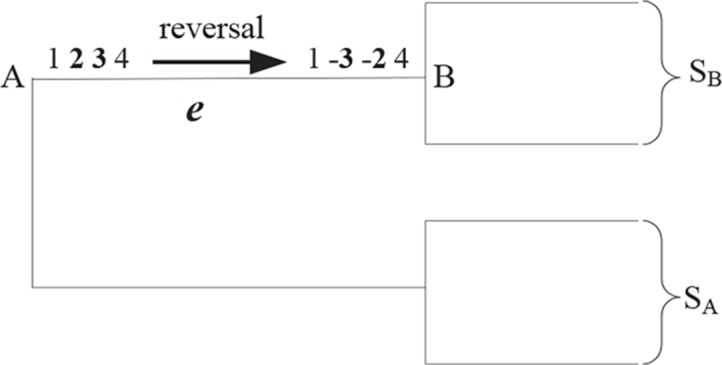
A reversal ***r*** (2, 3) during the evolution from *A* to *B*; *S*\s\do5 **(A)** and *S*\s\do5 **(B)** are two subsets of all leaves species divided by the edge *e*.

**Figure 1** shows a reversal *r*(2, 3) during the evolution from *A* to *B*, where *A* and *B* are two uni-chromosomal genomes, and the chromosomes are *X =* 1,234 and *Y =* 1 – 3 – 24, respectively. The set of genomes will be divided into two subsets recorded by *S_A_* and *S_B_* after removing the edge *e* from *T*. Suppose there is not any rearrangement events inside *S_A_* and *S_B_*. Then, adjacencies *a*(1, 2) and *a*(3, 4) can be found in each genome of *S_A_* and not in any one genome of *S_B_*; *a*(1,−3) and *a*(−2,4) can be found in each genome of *S*_B_ and not in any one genome of *S_A_*. In turn, we can utilize the four adjacencies *a*(1, 2), *a*(3, 4), *a*(1, −3), and *a*(−2,4) to identify a reversal *r*(2, 3) occurring on the edge *e*. The EMRAE method infers the rearrangement events by means of the similar rules.

Let *e* = (*A*, *B*) be an edge of *T*, *G*={*G*_1_,*G*_2_,⋯,*G _m_*}the genomes of leaves, and *a*_1_,*a*_2_,⋯*a _i_* the children of *A* and *b*_1_,*b*_2_,⋯*b*_j_ the children of *B*. EMRAE first selects a number of adjacencies as candidate adjacencies *Ca*(*e*,*A*) for edge *e* and node *A* according the following steps.

1. Find the adjacencies are in each genome of *S _A_* and not in any one genome of *S _B_*, then put them to *Ca*(*e*, *A*);
2. If *A* is an internal node, find all edges connected with *A* except *e* and record them with *e*_1_,*e*_2_,⋯,*e _k_*. For each *e _i_*=(*u _i_*,*A*)(1≤*i*≤*k*), *G* can be divided into two parts after removing *e _i_*, *S _ui_* is the part not including *A*.
  - Find the adjacencies that are in one genome of each S_ui_ (1 ≤ i ≤ k) and not in any one genome of *S _B_*, then put them to *Ca*(*e*,*A*);
  - Compute *Ca*(*e _i_*, *u _i_*) and *Ca*(*e _i_*,*u*)(1≤*i*≤*k*). For each one *Ca*(*e _i_*, *u _i_*), find the adjacency *a*_1_ from *Ca*(*e _i_*, *u _i_*), such that *a*_1_ is not overlap gene with any one adjacency in *Ca*(*e _i_*, *u*), *a*_1_ has overlap gene with one adjacency *a*_2_ in each *Ca*(*e _j_*,*u _j_*)(1≤*j*≠*i*≤*k*), and *a*_2_ has overlap gene with at least one adjacency in *Ca*(*e _j_*, *u*), then put *a*\s\do5(1) to *Ca*(*e*, *u*).

EMRAE then infers rearrangement from *Ca*(*e*, *A*) and *Ca*(*e*, *B*) for edge *e =* (*A*, *B*) with the help of inference rules in the following section. From the definitions of genome rearrangements, we find that each genome rearrangement can change several adjacencies. For example, each reversal *r*(*i*, *j*)(*i* ≤ *j*) can change two adjacencies *a*_1_=*a*(*c _i_*_–1_,*c _i_*) and *a*_2_=*a*(*c _j_*,*c _j_*_+1_) into *b*_1_ = *a*(*c _i_*–_1_, – *c _j_*) and *b*_2_=*a*(−*c _i_*,*c _j_*_+1_). Based on those facts, we obtain the inference rules introduced in the following section.

### Inference Rule

Let *e =* (*A*,*B*) be an edge of the phylogenetic tree *T*. Given adjacencies *a*_1_ = *a* (*c*_1_–_1_, *c _i_*), *a*_2_ = *a* (*c _j, _c_j_*_+1_) in *Ca*(*e*,*A*) and *b*_1_=*a*(*c _i_*_–1_,−*c _j_*), *b*_2_=*a*(−*c _i_*,*c _j_*_+1_) in *Ca*(*e*,*B*), EMRAE infers a reversal *r*(*i*,*j*) from *A* to *B* if all genomes are uni-chromosomal or *a*_1_, *a*_2_ are in the same chromosome in *S _A_* and *b*_1_, and *b*_2_ are in the same chromosome in *S _B_*. Otherwise, we infer a translocation *tl*(*i*, *j*). Similarly, given adjacencies *a*_1_=*a*(*c _i_*_–1_,*c _i_*), *a*_2_=*a*(*c _j_c_j_*_+1_) in *Ca*(*e*,*A*) and *b*_1_=*a*(*c _i_*_+1_,*c _j_*_+1_), *b*_2_=*a*(*c _j_*,*c _i_*) in *Ca*(*e*,*B*), EMRAE infers a translocation *tl*(*i*,*j*), or a reversal for *a*_1_, *a*_2_ in *Ca*(*e*,*A*) and adjacencies *b*_1_, *b*_2_ in *Ca*(*e*,*B*).

Assume that there are adjacencies *a*_1_=*a*(*c _i_*_–1_,*c _i_*), *a*_2_=*a*(*c _j_*,*c _j_*_+1_), and *a*_3_=*a*(*c _k_*,*c _k_*_+1_) in *Ca*(*e*,*A*) and *b*_1_=*a*(*c _i_*_–1_,*c _j_*_+1_), *b*_2_=*a*(*c _k_*,*c _i_*), and *b*_3_=*a*(*c _j_*,*c _k_*_+1_) in *Ca*(*e*,*B*). EMRAE can predict a transposition *tp*(*i*,*j*,*k*) during the evolution from *A* to *B* if all genomes are uni-chromosomal. Otherwise, suppose *m* genomes in *S _A_* have *a*_1_ and *a*_2_, then EMRAE can predict a transposition *tp*(*i*,*j*,*k*) if there are at least *m*/2 genomes such that the four integers of *a*_1_ and *a*_2_ on the same chromosome, or there are at least *m*/2 genomes such that the four integers of *a*_2_ and *a*_3_ on the same chromosome.

Assume that there is *a*=*a*(*c _i_*,*c _j_*) in *Ca*(*e*,*A*). EMRAE can predict a fission that splits the adjacency *a*=*a*(*c _i_*,*c _j_*) if *a* is sign-compatible for each genome *G _k_* in *S _B_*. The fusion from *A* to *B* can be seen as a fission from *B* to *A*.

### Recovering the Genome Rearrangements Based on Phylogenetic Tree

**Figure 2:**
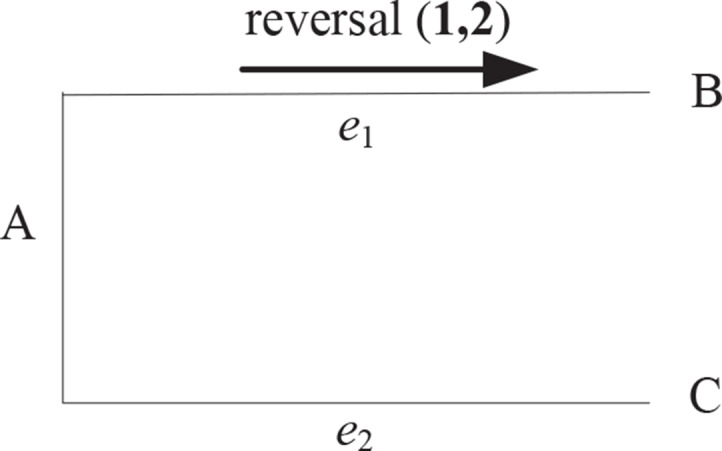
The tree topology with two taxa (**B** and **C**).

EMRAE can not identify the rearrangement occurring in the frontier of genomes. We take **Figure 2**, for example, where species *A*, *B*, and *C* are uni-chromosomal genomes *A =* 1,234, *B =*−2 – 134, and *C =* 1,234. A reversal r(1,2) has occurred in the evolution from *A* to *B*. EMRAE can compute the candidate adjacencies *a*(−1,3) for *Ca*(*e*_1_,*B*) and *a*(2,3) for *Ca*(*e*_1_,*A*). So, EMRAE can not infer the reversal r(1,2) on the edge *e*_1_ according to the candidate adjacencies.

We improve EMRAE so that the improved method (called RGRPT) is able to infer the rearrangement events occurring in the frontier region. The inference rule of RGRPT is the same as that of EMRAE. The difference between RGRPT and EMRAE is that they have different candidate adjacencies. RGRPT puts 0 to the head and tail for each chromosome, so there will be added a lot of adjacencies for each genome. For example, considering the uni-chromosomal genomes *X =* 1,234 and *Y =* −2 −134, the two additional candidate adjacencies *a*(0,1) and *a*(0,−2) are added.

RGRPT adds candidate adjacencies in the step b of EMRAE. For each one *Ca*(*e _i_*,*u _i_*) and an adjacency *a*_1_ from *Ca*(*e*_i_,*u*_i_), if there is an adjacency *a*_2_ in each *Ca*(*e _j_*,*u _j_*)(1≤*j*≠*i*≤*k*) such that *a*_1_ with *a*_2_ has overlap gene, then put *a*_1_ to *Ca*(*e*,*u*).

## Results

All of the experiments were performed on a computer with Intel Vostro 14 2.0 GHz CPU, 4 GB RAM, and 500 GB Hard Disk Drives (HDD). The operating system was Win10 64 bit with Java 1.6 installed. RGRPT was written in Java.

We tested RGRPT with both simulated data and the practical data (i.e., real biological data) introduced by the following section.

### Simulated Data

**Figure 3:**
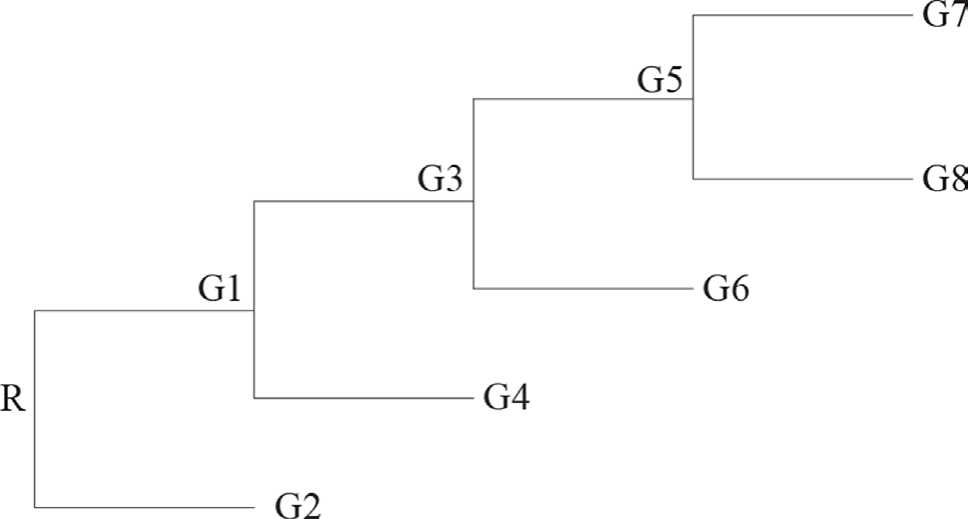
The topology used to generate the simulation data.

Here, we start with an uni-chromosomal genome as the ancestor, and it evolves along the phylogenetic tree with *n* taxa whose topology sees the **Figure 3**.

We generate two simulated data sets in order to test the affectivity of RGRPT. One of them is created from the phylogeny only with reversals events. The other data set is generated from the phylogeny with kinds of events, including reversals, translocation, transposition, fusion, and fission, and the quantity of those events is in a certain ratio. The two data sets can test the ability of methods to recover the simple and the complex evolution histories. First data set is created just using reversal events. Since the reversal on only one gene is rare (Korbel et al., 2007), we set the ratio of reversal on one gene and on more than one gene as 1:3. The number of leaves is from 3 to 10 with step 1. For each number of leaves, the ancestor genome with *m* gene, where *m* from 50 to 150 with step 10. Each edge will happen *k* reverse, where *k* is random integer number from 3 to 10. So, there are 11 groups data for each leaf number. Sensibility is the percentage of correctly predicted events in all practical events. Specificity is the percentage of correctly predicted events in all predicted events. We compute the sensibility and specificity for RGRPT and EMRAE for each group data. **Table 1** shows the average sensitivity and specificity for each leaf number. The second column of the table records the number of all events, and its last row records the average values.

**Table 1:** Results of EMRAE and recovering the genome rearrangements based on phylogenetic tree algorithms in predicting reversal events.

Leaves	Reversal	Sensibility		Specificity	
		EMRAE	RGRPT	EMRAE	RGRPT
3	24	64%	76%	89%	90%
4	39	65%	76%	94%	94%
5	45	61%	72%	92%	93%
6	59	57%	66%	90%	90%
7	69	54%	65%	92%	91%
8	79	59%	80%	92%	92%
9	92	55%	63%	90%	90%
10	104	55%	62%	89%	89%
Mean		58.7%	70%	91%	91.1%

**Table 1** shows that RGRPT achieves higher sensibility than EMRAE, and RGRPT achieves comparable specificity with EMRAE. Obviously, RGRPT can distinguish more actually occurred events than EMRAE. So, the experimental results show that the RGRPT is more efficient than EMRAE for predicting reversal events.

Second data set is generated by using all events, i.e., reversal, translocation, transposition, fusion, and fission. The reversals are generally more than the other rearrangement events. The fusions and the fissions are very rare; so, we record the number of the two events together. Here, we set the ratio of those events as 10:2:2:0.1. The ancestor genome has 5 chromosomes and each chromosome with 100 genes. The ancestor genome evolves along the topology with four leaves (see **Figure 3**). Each edge happen *k* events, where *k* is random number from 1 to μ and μ is 6, 12, 18, and 24. For each μ, it runs 10 times; so, we can obtain 10 groups data for each μ. **Table 2** shows the average of 10 groups data for each μ. This table indicates that the RGRPT is more efficient than EMRAE for predicting all events.

**Table 2:** Results of EMRAE and recovering the genome rearrangements based on phylogenetic tree algorithms in predicting all events.

Events of each edge	All events	Sensibility		Specificity	
		EMRAE	RGRPT	EMRAE	RGRPT
6	19	75.8%	85.7%	95.8%	96.2%
12	29	74.2%	80.3%	97%	96.5%
18	38	53.5%	58.1%	95.4%	96.7%
24	50	47.7%	50.5%	94.9%	94.1%
Mean		62.8%	68.7%	95.8%	95.9%

### Practical Data

**Figure 4:**
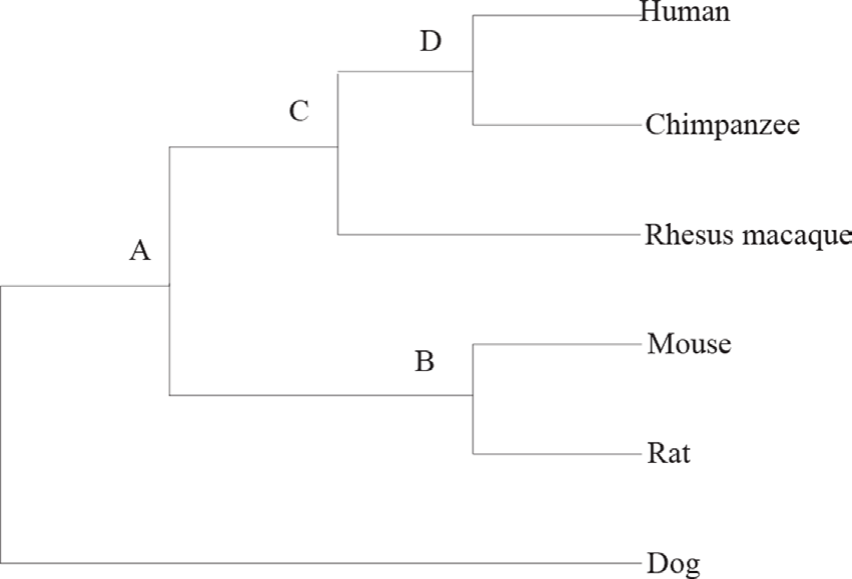
The tree describing the phylogeny of mammalian species.

The practical data is from the paper (Zhao and Bourque, 2009). It contains six mammalian genomes, i.e., human, chimpanzee, rhesus monkey, mouse, voles, and dog. The data are created from two different levels of resolution 10 kb and 50 kb. **Figure 4** is the tree describing the phylogeny of species. The results are shown in **Tables 3** and **4**. EM and RG represent EMRAE and RGRPT respectively, and Rev, Tloc, Tran, Fus, and Fis represent reversal, translocation, transposition, fusion, and fission, respectively. Each row in the table records the ancestor rearrangement events of the edge. For example, the values in the human row are the rearrangement events from D to human; the values in MR row are the rearrangement events from A and B.

**Table 3:** Genome rearrangement predictions of EMRAE and recovering the genome rearrangements based on phylogenetic tree at 10 kb resolution.

Species	Rev		Tloc		Tran		Fus/Fis		Total events	
	EM	RG	EM	RG	EM	RG	EM	RG	EM	RG
Human	12	13	0	0	4	5	0	0	16	18
HC	29	32	0	0	15	15	0	1	44	48
HCP	83	84	0	0	8	10	2	8	93	102
Chimp	17	19	0	0	7	8	1	1	25	28
Rhesus	49	50	0	0	40	42	1	2	90	94
Mouse	90	95	3	3	10	13	5	5	108	116
Rat	227	233	0	0	127	129	3	3	357	365
MR	140	143	2	3	9	10	0	0	151	156
Dog	184	189	10	10	17	17	14	14	225	230
Total	831	858	15	16	237	249	26	34	1,109	1,157

**Table 4:** Genome rearrangement predictions of EMRAE and recovering the genome rearrangements based on phylogenetic tree at 50 kb resolution.

Species	Rev		Tloc		Tran		Fus/Fis		Total events	
	EM	RG	EM	RG	EM	RG	EM	RG	EM	RG
Human	2	2	0	0	1	1	0	0	3	3
HC	19	19	0	0	4	4	1	1	24	24
HCP	27	29	0	0	5	6	2	6	34	41
Chimp	17	19	0	0	7	8	1	1	25	28
Rhesus	22	23	0	0	6	7	1	3	29	33
Mouse	25	27	3	3	0	0	5	6	33	36
Rat	128	131	0	0	65	65	5	5	198	201
MR	41	42	2	2	2	2	0	0	45	46
Dog	46	47	7	8	8	8	13	14	74	77
Total	322	332	12	13	92	94	28	36	454	475

At 10 kb resolution, the RGRPT algorithm predicts 1,157 ancestor rearrangement events, including 858 reversals, 16 translocations, 249 transpositions, and 34 fusions and fissions. It identifies 48 rearrangement events more than the EMRAE. The reversal events are in the majority in all predicted events. At 50 kb resolution, the RGRPT algorithm predicts 475 ancestor rearrangement events, including 332 reversals, 13 translocations, 94 transpositions, and 36 fusion and fissions. RGRPT identifies 21 rearrangement events more than EMRAE algorithm. The rearrangement events identified in the rat edge are mostly in all edges either at 10 kb resolution or at 50 kb resolution. The syntenic blocks of genomes at 10 kb resolution are more than the syntenic blocks of genomes at 50 kb resolution. The fact reduces the recognized rearrangement events at 10 kb resolution that are more than the recognized rearrangement events at 50 kb resolution. Experiments show that RGRPT can recover more ancestor events than EMRAE.

## Discussion

This paper proposes a new method, RGRPT, to infer ancestor rearrangement events. RGRPT takes a phylogenetic tree describing the evolution of species and the genomes of species as input. Experiments on the simulated data and practical data show that RGRPT is more efficient than EMRAE and can recover more ancestor rearrangement events than EMRAE. RGRPT provides a method for us to research the genome rearrangement of species. We can use RGRPT to recognize the ancestral genome rearrangement for the evolution of other species in future (Tian et al., 2018).

## Data Availability Statement

Publicly available datasets were analyzed in this study. These data can be found here: https://github.com/wangjuanimu/data-of-genome-rearrangement.

## Author Contributions

JW proposed and implemented the RGRPT method. JW and BC designed all experiments. All authors participated in the designing the algorithm and writing the paper.

## Funding

The work was supported by the National Natural Science Foundation of China (61661040, 61661039, 61571163, 61532014, 61671189, 91735306, 61751104); the National Key Research and Development Plan Task of China (Grant No. 2016YFC0901902).

## Conflict of Interest

The authors declare that the research was conducted in the absence of any commercial or financial relationships that could be construed as a potential conflict of interest.
